# Contribution of respiratory tract infections to child deaths: a data linkage study

**DOI:** 10.1186/1471-2458-14-1191

**Published:** 2014-11-20

**Authors:** Pia Hardelid, Nirupa Dattani, Mario Cortina-Borja, Ruth Gilbert

**Affiliations:** Population, Policy and Practice Programme, UCL Institute of Child Health, 30 Guilford Street, London, WC1N 1EH UK; Centre for Maternal and Child Health Research, City University, London, UK

**Keywords:** Respiratory tract infection, Mortality, Children, Data linkage

## Abstract

**Background:**

Respiratory tract infections (RTIs) are an important cause of death in children, and often contribute to the terminal decline in children with chronic conditions. RTIs are often underrecorded as the underlying cause of death; therefore the overall contribution of RTIs to child deaths and the potential preventability of RTI-related deaths have not been adequately quantified.

**Methods:**

We analysed deaths in children resident in England who died of non-injury causes aged 28 days to 18 years between 2001 and 2010 using death certificates linked to a longitudinal hospital admission database. We defined deaths as RTI-related if RTIs or other respiratory conditions were recorded on death certificates or linked hospital records up to 30 days before death. We examined trends in mortality by age group, year and season (winter or summer) and determined the winter excess of RTI-related deaths using rate differencing techniques. We estimated the proportion of RTI-related deaths in children with chronic conditions.

**Results:**

22.4% (5039/22509) of child deaths were RTI-related. RTI-related deaths declined by 2.3% per year in infants aged 28 to 364 days between 2001 and 2010. No decline was observed for older children. On average there were 161 winter excess RTI-related deaths annually, accounting for 32% of all RTI-related deaths. 89.0% of children with RTI-related deaths had at least one chronic condition; neurological conditions were the most prevalent.

**Conclusions:**

RTI-related deaths have not declined in the last decade except in infants. Targeted strategies to prevent the winter excess of RTIs and to treat RTIs in children, particularly children with chronic conditions, may reduce RTI-related deaths.

**Electronic supplementary material:**

The online version of this article (doi:10.1186/1471-2458-14-1191) contains supplementary material, which is available to authorized users.

## Background

Respiratory tract infections (RTIs) are an important contributor to mortality in children worldwide [1–3]. Childhood mortality from respiratory conditions in England and Wales declined by 85% to 92% between the 1960s and the 1990s [4]. Yet, research into the mortality burden of specific pathogens have shown that influenza and respiratory syncytial virus (RSV) are estimated to cause up to 500 deaths in children in England and Wales every year [5, 6]. This is despite national influenza and passive RSV vaccination programmes targeted at children deemed at high risk [7]. In 2009/10, three years after the introduction of seven-valent pneumococcal vaccine, 17 deaths per year were estimated to occur due to invasive pneumococcal disease in young children in England [8]. Few studies have examined the overall contribution of RTIs to childhood mortality, despite the importance to policy.

Quantifying the mortality burden of RTIs is not straightforward. Many previous studies rely on death certification data alone, however difficulties in accurately determining and recording causes of death are well described [9–11]. A further complication is that RTI-related deaths often occur in people with complex health problems, in whom RTIs contribute to the terminal decline [12]. It can therefore be difficult to determine whether the RTI was a cause or a consequence of the underlying health condition. This particularly becomes an issue when attempting to infer what proportion of RTI deaths are preventable through improvements in care or public health interventions such as vaccination.

In this study we use coded clinical information from death certificates linked to electronic hospital admission records from all non-injury deaths in children to define deaths related to RTIs. Although the preventability of RTI-related deaths cannot be directly estimated using administrative data, we inferred preventability by looking at trends over time in RTI-related deaths, contrasting with trends in overall non-injury mortality, and estimating excess RTI-related deaths in winter. We also examine the proportion of RTI-related deaths that occur in children with chronic conditions.

## Methods

### Data sources

We analysed death certificates linked to a longitudinal hospital admission database for all resident children in England who died of non-injury related causes, based on their underlying cause of death, aged between 28 days and 18 completed years between January 2001 and December 2010. Linkage was carried out by the Trusted Data Linkage Service of the Health and Social Care Information Centre, using a deterministic algorithm based on NHS number, date of birth, sex and postcode. The linkage methods and accuracy are described in detail elsewhere [13]. Upon receipt of the data, we checked and cleaned them according to algorithms described previously [14]. The dataset included late-registered deaths until 7th August 2012. Hospital admission records were obtained from the Hospital Episode Statistics (HES) database [15] and included any admissions to National Health Service (NHS) hospitals in England from April 1997. Death certificates and hospital records were coded using the International Classification of Diseases version 10 (ICD-10). Information on prescribing is not available in HES. Population denominators by age and year were obtained from the Office for National Statistics.

### Ethics

All identifiers used for linkage were removed before data was sent to us. The data we used for the study were therefore anonymised, without personal identifiers. The data were collected as part of routine clinical practice or vital registration processes, not for research. Following guidance from the NHS Health Research Authority on using anonymised data collected as part of routine clinical practice for research purposes, [16] we did not seek ethical review of this study.

### Definition of RTI-related deaths and chronic conditions

Since RTIs are likely to be variably coded in administrative health databases, we chose a broad definition of RTI-related deaths to minimise undercounting. We identified RTI-related deaths using ICD-10 codes A37, J00-J22, which are specific for acute RTIs, based on codes listed anywhere on death certificates, or hospital discharge records where the date of the start of the hospital episode (an episode is a period of continuous care under one consultant [15]) was up to 30 days before death. We also conducted secondary analyses using a more sensitive cluster of codes indicating any respiratory condition, including RTIs (ICD-10 A37, J00-J99, R05, R06, E84, P75, Q30-Q34, Q790, G47.3, P22-P28), in children who died. The estimate of winter excess deaths related to any respiratory condition defined by the sensitive coding cluster could be interpreted as an upper bound on the number of excess RTI-related deaths.

Chronic conditions were defined as any condition that would require medical follow-up for one year or more, based on ICD-10 codes on death certificates or in hospital records up to one year before death [14]. Chronic conditions were grouped into eight categories for analyses: mental/behavioural, cancer/blood, chronic infection, respiratory, endocrine/metabolic/renal/digestive/genitourinary, musculoskeletal/skin, neurological/sensory and cardiac conditions.

### Statistical analyses

All analyses were carried out according to age group at death (28 to 364 days, one to four years and five to 18 years). We calculated annual RTI-related mortality rates to examine trends over time. Poisson regression models were fitted with year as a linear term to determine whether any observed trends in annual mortality rates were statistically significant (defined as a likelihood ratio test *p* <0.05) comparing an intercept only model and a model including year). If the trend was significant, possible change points were examined using piecewise generalised linear models to determine the change point location [17]. Since these models were not nested, we used the Akaike Information Criterion (AIC) to determine whether a model including a change point improved the overall fit. We compared trends in RTI-related mortality rates to those for all non-injury causes.

We assumed that some of the winter excess of RTI-related deaths could potentially be prevented through prompt identification and treatment (including with antibiotics), effective management of chronic conditions (such as enhanced care for children with breathing support), universal vaccination strategies with effective vaccines, or improved hygiene measures such as hand washing. To examine seasonality we plotted weekly and monthly mortality rates per 100,000 population. Monthly rates were plotted using circular plots, using the season library in R [18]. RTI mortality rates in winter (defined as week 40 to week 20 the following year) and summer (week 21 to week 39) were calculated for the whole study period, adjusting the population denominator to account for the differing person-time at risk. This definition of a winter period was chosen since it covers the weeks during which respiratory infection surveillance programmes are active in England [19]. We estimated the number of excess RTI-related deaths in winter periods compared with summer periods using rate differencing techniques [6, 20]. Further details can be found in the Additional file 1. We calculated the number of excess RTI-related deaths in winter as a percentage of all RTI-related deaths, and as a percentage of all non-injury deaths occurring in winter.

We calculated the proportion of RTI-related deaths that occurred in children with chronic conditions, according to type of chronic condition and age group. Denominators for children with and without chronic conditions were not available. Therefore we could not use rate differencing techniques to estimate the number of excess RTI-related deaths according to chronic condition type. Instead, we compared the proportion of all RTI-related deaths which occurred in winter in children with and without chronic conditions respectively. We used *χ*^*2*^ tests to test for differences in the proportions of RTI-related deaths occurring in children with chronic conditions. These analyses were carried out using the RTI-specific code lists to define RTI-related deaths.

Methods and results of sensitivity analyses are given in the Additional file 1. All statistical analyses were carried out in R version 3.0.1 [21].

## Results

The study population comprised 22509 children who died of non-injury causes between 2001 and 2010, of whom 9208 were aged between 28 and 364 days (40.9%), 4310 one to four years (19.1%) and 8991 five to 18 years (39.9%). 14180 children (63.0%) were linked to at least one hospital record within the last thirty days before death.

Overall, 3339 children who died of non-injury causes had an RTI listed on their death certificate (14.8%), and 5039 children (22.4%) had an RTI listed on death certificates or on hospital records up to 30 days before death. 10169 children (45.2%) had at least one respiratory condition (including RTIs and other respiratory conditions) listed on their death certificates or on hospital records up to 30 days before death. This was equivalent to 504 RTI-related deaths and 1017 any respiratory condition-related deaths per year respectively. 18443 children had at least one chronic condition (81.9%).

Recording of RTIs and respiratory conditions on death certificates and hospital records was generally non-specific (Additional file 1: Table S1), that is, not mentioning specific pathogens. There was weak agreement between hospital records and death certificates in recording of any respiratory condition (Additional file 1: Table S2); with only 66.1% agreement for children who died in hospital. Mortality rates for RTIs and other respiratory conditions were between two and eight times higher when including all causes listed on death certificates and linked hospital records up to one month before death compared to using the underlying cause only (Additional file 1: Table S3).

### Annual trends in mortality rates from RTIs

**Figure 1:**
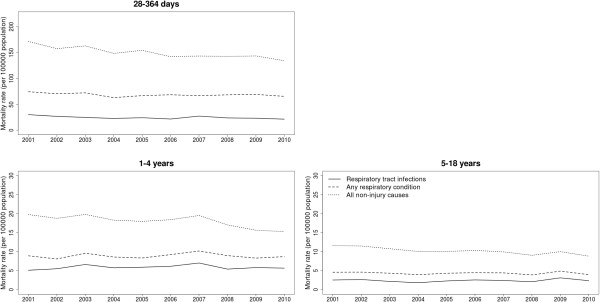
**Mortality rates (per 100,000 population) for RTIs, any respiratory condition and all non-injury causes by age group, 2001–2010.** Note different y-axis scales for children aged less than one year.

Results from the Poisson regression models showed that RTI-related deaths declined significantly between 2001 and 2010 by an average of 2.3% per year (95% CI 0.6%, 4.0%) in children aged between 28 and 364 days, as shown in Figure 1 (LR test *p* = 0.01 for year as a linear trend compared to intercept only model). This amounted to a total decline of 28.9% between 2001 (a mortality rate of 30.1/100,000 population) and 2010 (when the mortality rate had declined to 21.4/100,000 population). We detected a change point in the series in 2003, prior to which the decline was more pronounced. No significant declines were observed for RTI-related deaths in older children. No trends were observed for deaths mentioning any respiratory condition in any age group. In contrast, non-injury all-cause mortality rates showed significant annual declines of 2.2% in children aged 28 to 364 days, 2.4% in one to four year olds and 2.6% in five to 18 year olds during the same period (Figure 1).

### Estimation of excess winter RTI deaths

**Figure 2:**
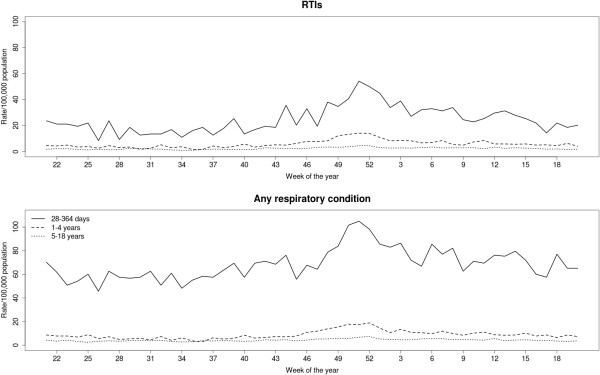
**Mortality rates (per 100,000 population) by week of the year, age group for deaths involving RTIs and any respiratory condition.**

**Figure 3:**
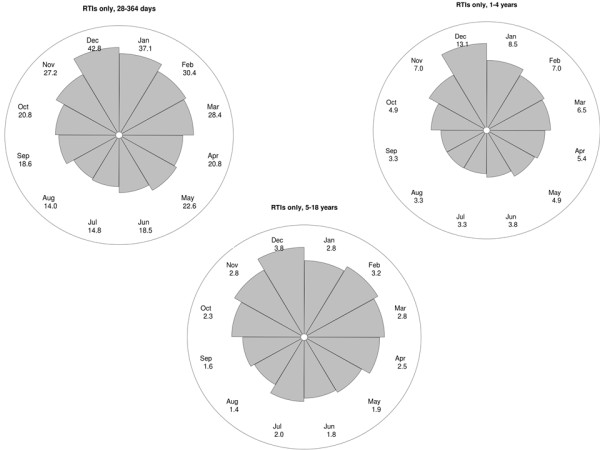
**Circular plot of monthly RTI-related mortality rates by age group (per 100,000 population).** The distance from the centre of the circle to the edge is proportional to the mortality rate.

**Figure 4:**
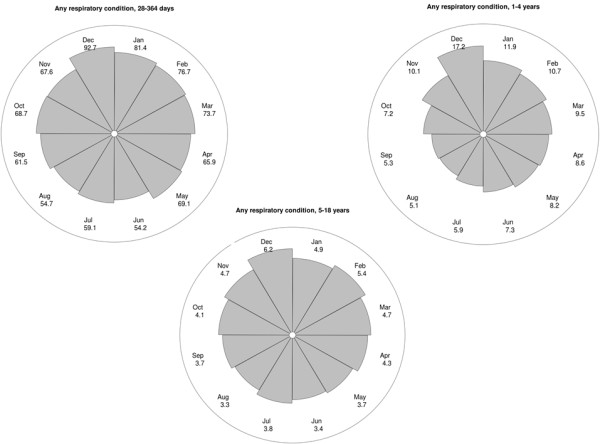
**Circular plot of monthly mortality rates for any respiratory condition by age group (per 100,000 population).** The distance from the centre of the circle to the edge is proportional to the mortality rate.

A seasonal pattern was observed for mortality involving respiratory conditions, particularly for RTI-related deaths (Figure 2 and Table 1). Mortality rates peaked in December for both specific RTI-related mortality and for deaths involving any respiratory condition, but seasonal variation in respiratory mortality was particularly evident in one to four year old children (Figures 3 and 4).

**Table 1:** **Mortality rates by season, rate differences (both per 100,000 population), and rate ratios comparing winter and summer, for children with RTIs only and any respiratory condition, with winter excess deaths shown as estimated number and percentage of all non-injury winter deaths and all RTI-related deaths, by age group and indicator used, England, 2001-2010**

Indicator used	Rate _winter_(95% CI)	Rate _summer_(95% CI)	Rate ratio _winter/summer_(95% CI)	Rate difference _winter-summer_(95% CI) ^†^	Total number of excess RTI-related deaths in winter (range)*	Average number of excess RTI-related deaths each winter (range)*	Winter excess as % of total non-injury deaths in winter**	Winter excess as % of total RTI-related deaths***
	**28-364 days**							
**RTIs only**	28.7 (27.1, 30.5)	17.2 (15.5, 19.0)	1.68 (1.49, 1.88)	11.59 (9.19, 13.98)	454 (360, 548)	45 (36, 55)	7.3%	30.0%
**Any respiratory condition**	74.4 (71.7, 77.1)	58.1 (54.9, 61.3)	1.28 (1.20, 1.37)	16.23 (12.08, 20.38)	636 (474, 799)	64 (47, 80)	10.2%	15.1%
	**1-4 years**							
**RTIs only**	7.3 (6.8, 7.7)	3.5 (3.1, 3.9)	2.08 (1.84, 2.37)	3.78 (3.20, 4.36)	576 (487, 664)	58 (49, 66)	18.8%	40.8%
**Any respiratory condition**	10.5 (10.0, 11.0)	6.1 (5.6, 6.7)	1.71 (1.55, 1.89)	4.37 (3.63, 5.10)	665 (553, 776)	66 (55, 78)	21.8%	31.2%
	**5-18 years**							
**RTIs only**	2.8 (2.6, 2.9)	1.7 (1.6, 1.9)	1.60 (1.45, 1.76)	1.04 (0.84, 1.24)	583 (471, 695)	58 (47, 69)	9.6%	27.6%
**Any respiratory condition**	4.8 (4.6, 4.9)	3.6 (3.4, 3.8)	1.32 (1.23, 1.41)	1.14 (0.87, 1.42)	640 (485, 794)	64 (49, 79)	10.6%	16.7%
	**All ages**							
**RTIs only**	5.0 (4.9, 5.2)	2.9 (2.7, 3.1)	1.74 (1.63, 1.86)	2.1 (1.9, 2.4)	1613 (1442, 1784)	161 (144, 178)	10.5%	32.0%
**Any respiratory condition**	9.6 (9.3, 9.8)	7.0 (6.7, 7.2)	1.37 (1.31, 1.43)	2.6 (2.3, 2.9)	1941 (1691, 2191)	194 (169, 219)	12.6%	19.1%

^**†**^All differences in RTI-related mortality rates between winter and summer are statistically significant, Likelihood ratio test *p* < 0.001 for all ages.

*Range is estimated using the lower and upper end of the 95% confidence interval for the rate difference.

**Using point estimate for excess RTI-related deaths. Total non-injury deaths in winter by age group: 28–264 days = 6244, 1–4 years = 3056, 5–18 years = 6046.

***Using point estimate for excess RTI-related or any respiratory condition related deaths.

Total deaths by age group for RTIs only: 28–264 days = 1512, 1–4 years = 1411, 5–18 years = 2116.

Total deaths by age group for any respiratory condition: 28–264 days = 4219, 1–4 years = 2129, 5–18 years = 3821.

There were 161 excess winter RTI-related deaths in children aged between 28 days and 18 years each year (Table 1), accounting for 32% of all RTI-related deaths. If the more sensitive indicator, excess deaths with any respiratory condition mentioned, was used, up to 194 excess winter RTI-related deaths occurred annually. Although the highest RTI-related mortality rates were observed in children aged less than one year, excess winter RTI-related deaths accounted for up to 41% of the total number of RTI-related deaths and up to 22% of all winter deaths in one to four year olds (Table 1).

### RTI-related deaths in children with chronic conditions

Among children whose deaths were RTI-related, 89.0% of 5039 children had one or more chronic conditions, compared to 79.9% of 17470 children who did not die with an RTI (*χ*^*2*^ test *p* <0.001). The proportion of children whose deaths were RTI-related who also had a chronic condition increased with age. 79.3% of children aged between 28 and 364 days (of a total of 1512 children), 89.9% of children aged one to four years (1411 children) and 95.3% of five to 18 year old children (2116 children) who died with RTIs had a chronic condition (*χ*^*2*^ test for trend *p* <0.001). Neurological/sensory conditions were the most common chronic conditions among children who died with RTIs; 64.6% of children whose deaths were RTI-related had a neurological sensory condition (Table 2). Of the 4483 children with one or more chronic conditions whose death was RTI-related, 75.2% had chronic conditions from two or more of the eight chronic condition groups.

**Table 2:** Number and percentage (in brackets) of children who died with RTIs*, by type of chronic condition and age group

	Mental/behavioural	Cancer/blood	Chronic infections	Respiratory	Endocrine/metabolic/renal/digestive/genitourinary	Musculoskeletal/skin	Neurological/Sensory	Cardiac
**28-364 days**	33 (2.2)	161 (10.6)	36 (2.4)	554 (36.6)	487 (32.2)	208 (13.8)	799 (52.8)	544 (36.0)
***n*** **= 1512**								
**1-4 years**	229 (16.2)	257 (18.2)	38 (2.7)	623 (44.2)	642 (45.5)	227 (16.1)	952 (67.5)	339 (24.0)
***n*** **= 1411**								
**5-18 years**	530 (25.0)	496 (23.4)	68 (3.2)	889 (42.0)	870 (41.1)	575 (27.2)	1505 (71.1)	315 (14.9)
***n*** **= 2116**								
**All ages**	792 (15.7%)	914 (18.1)	142 (2.8)	2066 (41.0)	1999 (39.7)	1010 (20.0)	3256 (64.6)	1198 (23.8)
***n*** **= 5039**								

*RTI-related deaths are defined using the following ICD-10 codes: A37, J00-J22.

Note that percentages will add up to more than 100% since chronic condition groups are not mutually exclusive and children can have conditions from more than one group.

Of the 4483 children who died with RTIs and chronic conditions, 74.7% (3349 children) died in winter. This was significantly lower than among children who died with RTIs but without chronic conditions, among whom 79.7% (443 of 556 children) died in winter (*χ*^*2*^ test *p* = 0.01), although the absolute difference in proportions was small.

## Discussion

There has been no decline in RTI-related mortality in children aged one year and above since 2001, despite significant declines in all-cause non-injury deaths. We estimated that an average of 161 excess RTI-related deaths occurred each winter in children aged 28 days to 18 years in England. This excess accounts for 32% of all RTI-related deaths. Although the highest mortality rates from RTIs in winter were found in children aged between one and 11 months, children aged between one and four years experienced the highest number of excess winter RTI-related deaths as a proportion of all RTI-related deaths. 89% of children who died from RTIs had a chronic condition; 65% had a neurological/sensory condition.

This was a large study of all deaths in children aged 28 days to 18 years in England during a ten year period. Since deaths from RTIs are relatively rare in children compared to the elderly, the population wide coverage of the study was necessary to ensure sufficient cases to estimate the number of RTI-related deaths with reasonable precision.

We used linked data involving death certificates and hospital admissions which allowed identification of deaths where an RTI was severe enough to warrant recording on a hospital database shortly preceding death, even if this was not recorded on death certificates. Use of linked hospital admission data increased the sensitivity of our analyses for detecting RTI-related deaths and the presence of chronic conditions, compared to using death certificates alone. Through linkage, we were able to identify a further 1700 children (amounting to 34% of the total number of cases) who had an RTI on their hospital record, but this was not mentioned on the death certificate.

We identified relatively poor agreement between hospital records and death certificates in recording of any respiratory condition (66.1% agreement for deaths in hospital and 73.2% agreement for deaths outside hospital). Poor agreement between hospital records and death certificates has been reported previously [22–24]. Data linkage provides a method of validating the diagnostic information recorded in these datasets, as well as a strategy to overcome some of the under-recording of diagnoses on death certificates.

The estimate of the proportion of children who had an RTI in the month before death is likely to be an underestimate, as linkage was limited to hospital records. Further linkage to primary care databases and to data on diagnoses and treatment provided in hospices would allow an examination of the role of RTIs in children who died but who had not had a hospital admission in the month before death. However, there are currently no national-level primary care or hospice datasets available in England.

We could not compare differential risks of RTI-related mortality according to type of chronic condition since we did not have information on all children (alive and dead) who were affected according to type of chronic condition. Future studies using longitudinal hospital data from birth in children are required. These can be used to estimate RTI-related death rates according to type of chronic condition and other determinants such as deprivation level, birth weight and gestational age.

Although our method allowed us to estimate the number of children whose deaths were RTI-related, it was not possible to determine whether a child died of an RTI. The exact chronology of events is difficult to infer from hospital databases, since dates of infection onset or diagnoses were not available. Further linkage to laboratory datasets would allow the quantification of deaths from specific pathogens and more detailed validation of diagnostic coding. This would also allow characterisation and subanalyses of community and hospital acquired infections.

We inferred preventability using trends over time and differences between winter and summer in RTI-related mortality rates. The rate differencing technique used to estimate excess winter RTI-related deaths was a crude attempt to estimate the number of deaths that may be preventable. However, the vast majority of children whose deaths were RTI-related had a chronic condition, and of them, 75% had conditions affecting more than one body system, indicating complex health problems. It is therefore unlikely that all of these deaths could be prevented, even through the use of interventions that may be highly effective in healthy children. In children with very complex health problems, it is possible that antibiotic treatment or influenza or pneumococcal vaccination may only delay death, not prevent the death completely. Such a ‘harvesting’ effect has been described in relation to the effect of extreme weather on respiratory mortality in adults, [25] but there is little evidence regarding such effects in children, most likely due to mortality being relatively rare at younger ages. In addition, it is likely that the number of excess winter deaths represents only a small proportion of all preventable RTI deaths throughout the year. Case note reviews of a subset of these deaths could help to determine whether an RTI was likely to be the cause of the death and whether an RTI-related death could have been delayed or prevented.

Previous studies have focused on estimating the mortality burden of particular pathogens, including influenza and RSV, using statistical models. Assuming the majority of RTI-related deaths in children are due to those two viruses, our results are broadly compatible with estimates of influenza and RSV related deaths in England [6], although our study included a later time period. The studies by Pitman et al. [26] and Hardelid et al. [5] are not directly comparable to ours as they also include neonates.

Chronic conditions have previously been recognised as risk factors for severe outcomes of RTIs in children, [27–30] but there is a lack of evidence regarding which interventions are effective for these children [27–30]. For example, Osterholm et al. [31] estimated that live attenuated influenza vaccine (now recommended for children aged two to four years in the UK) is 83% effective at preventing confirmed influenza infection in healthy children. However, there is scant evidence for vaccine effectiveness in children with chronic conditions, particularly for preventing deaths. Likewise, industry-funded trials have shown that palivizumab reduces hospital admissions for RSV in high risk children by 50%, but with no evidence of significant reductions in mortality [32]. Pneumococcal vaccine effectiveness has been estimated at 75%, [33] however since the case fatality rate for invasive pneumococcal disease is around 4% in children, [34] the effect on mortality is likely to be small.

## Conclusions

Over 500 RTI-related deaths occur in children in England each year. Up to one third of these are related to seasonal variation, and a subset may be preventable. Nearly 90% of RTI-related deaths occur in children with chronic conditions, and the highest incidence is in children aged less than one year. Effective implementation of prevention and treatment of RTIs is likely to lower the mortality burden in children, particularly in younger children aged less than five years. The use of routinely collected data offer the possibility of regular assessment of mortality rates to determine the mortality impact of RTIs and the effect of public health and treatment interventions.

### Availability of supporting data

Sharing of the raw data used for this study is not allowed under agreements with the data providers, due to the possibility of individual disclosure.

## Electronic supplementary material

Additional file 1: **Supplementary text and tables.** (DOCX 33 KB)

## Abbreviations

HES: Hospital episode statistics
ICD-10: International Classification of Diseases version 10
NHS: National Health Service
RSV: Respiratory syncytial virus
RTI: Respiratory tract infection.
