# Supplementary material for: Contribution of respiratory tract infections to child deaths: a data linkage study
Source: BMC Public Health. 2014 Nov 20;14:1191. doi: 10.1186/1471-2458-14-1191 (PMC4247691; doi:10.1186/1471-2458-14-1191)
Supplement: Supplementary file 1 — Additional file 1: Supplementary text and tables. (DOCX 33 KB) [file 12889_2014_7315_MOESM1_ESM.docx]

**SUPPLEMENTARY INFORMATION**

**METHODS**

**Definition of RTIs on hospital records within 30 days of death and cleaning of episode start dates**

We used the episode start date to determine whether an RTI diagnosis code was recorded within 30 days of the death date (where an episode is a period under the continuous care of one consultant). In cases where the episode start date was coded as either before the admission date or after the discharge date (188/433026 episodes), the episode start date was set to the admission date or the discharge date respectively.

**Agreement in recording between hospital records and death certificates**

We calculated the percentage agreement in recording of any respiratory condition (A37, J00-J99, R05, R06, E84, P75, Q30-Q34, Q790, G47.3, P22-P28) between hospital records and death certificates for deaths occurring during an admission (that is, where the difference between the date of death and the date of discharge was less than or equal to 0).

**Estimating excess RTI-related deaths in winter using rate differencing techniques**

We used rate differencing techniques to estimate excess RTI-related deaths in winter. This method estimates the number of excess RTI-related deaths during winter by multiplying the rate difference between RTI mortality rates in winter and summer by the person-time at risk in winter.[[1](#_ENREF_1) , [2](#_ENREF_2)] Excess deaths were only calculated if the differences in rates between winter and summer were statistically significant. We fitted Poisson regression models with counts of deaths as the outcome, person-time as an offset, and including a term for season. If the Likelihood Ratio test *p*-value for the season term was less than 0.05, we assumed the differences were statistically significant.

**Sensitivity analyses**

We validated the final linked datasets against a dataset of all deaths in children aged up to 18 years provided by the Office for National Statistics. Whilst the number of children aged one to 18 years was nearly identical in the two datasets (a difference of three children), children who died aged 28 to 364 days were undercounted in the linked dataset by 1.8%. We therefore carried out sensitivity analyses to determine the effect of this undercounting on the number of deaths from respiratory viruses.

The UK observed two peaks of H1N1 influenza circulation during the summer and autumn of 2009, and a small number of deaths in children were reported during the first wave which peaked in July.[[3](#_ENREF_3)] We therefore carried out sensitivity analyses where week 26 to week 33 of 2009 were included in the winter period.

**RESULTS**

**Codes used to record RTIs and other respiratory conditions on hospital records and death certificates**

Table S1. Ten most common codes listed on hospital records (up to 30 days before death) and on death certificates for respiratory tract infections and other respiratory conditions, children aged 28 days to 18 years, 2001-2010

| **Respiratory tract infections (RTIs)** | | | | | |
| --- | --- | --- | --- | --- | --- |
| **Hospital records** | | | **Death certificates** | | |
| **Code** | **Description** | **Number of times listed** | **Code** | **Description** | **Number of times listed** |
| J22 | Unspecified acute lower respiratory infection | 1137 | J189 | Pneumonia, unspecified | 1281 |
| J189 | Pneumonia,unspecified | 783 | J180 | Bronchopneumonia, unspecified | 1056 |
| J181 | Lobar pneumonia, unspecified | 750 | J22 | Unspecified acute lower respiratory infection | 163 |
| J069 | Acute upper respiratory infection, unspecified | 395 | J219 | Acute bronchiolitis, unspecified | 142 |
| J210 | Acute bronchiolitis due to respiratory syncytial virus | 292 | J209 | Acute bronchitis, unspecified | 112 |
| J219 | Acute bronchiolitis, unspecified | 220 | J09 | Influenza due to certain identified influenza virus | 79 |
| J180 | Bronchopneumonia, unspecified | 211 | J181 | Lobar pneumonia, unspecified | 58 |
| J101 | Influenza with other respiratory manifestations, other influenza virus identified | 80 | J069 | Acute upper respiratory infection, unspecified | 56 |
| J151 | Pneumonia due to Pseudomonas | 60 | J210 | Acute bronchiolitis due to respiratory syncytial virus | 56 |
| J100 | Influenza with pneumonia, other influenza virus identified | 48 | J111 | Influenza with other respiratory manifestations, virus not identified | 53 |

Table S1. Continued

| **Other respiratory condition (excluding RTIs)** | | | | | |
| --- | --- | --- | --- | --- | --- |
| **Hospital records** | | | **Death certificates** | | |
| **Code** | **Description** | **Number of times listed** | **Code** | **Description** | **Number of times listed** |
| J969 | Respiratory failure, unspecified | 830 | J969 | Respiratory failure, unspecified | 1185 |
| J90 | Pleural effusion, not elsewhere classified | 562 | P279 | Unspecified chronic respiratory disease originating in the perinatal period | 792 |
| P220 | Respiratory distress syndrome of newborn | 501 | J988 | Other specified respiratory disorders | 718 |
| R060 | Dyspnoea | 466 | J984 | Other disorders of lung | 425 |
| J960 | Acute respiratory failure | 457 | J690 | Pneumonitis due to food and vomit | 376 |
| J459 | Asthma, unspecified | 454 | J450 | Predominantly allergic asthma | 209 |
| R068 | Other and unspecified abnormalities of breathing | 404 | E849 | Cystic fibrosis, unspecified | 188 |
| P271 | Bronchopulmonary dysplasia originating in the perinatal period | 377 | P271 | Bronchopulmonary dysplasia originating in the perinatal period | 171 |
| J690 | Pneumonitis due to food and vomit | 369 | J80 | Adult respiratory distress syndrome | 145 |
| J984 | Other disorders of lung | 349 | J958 | Other postprocedural respiratory disorders | 142 |

**Agreement in recording between death certificates and hospital records**

Table S2. Agreement in recording of any respiratory condition on death certificates and hospital admission during the last 30 days before death in children: 2001-2010

| **Deaths occurring during an admission**  **Agreement=66.1%** | | **Recorded on death certificate** | | |
| --- | --- | --- | --- | --- |
|  |  | **Yes** | **No** | **Total** |
| **Recorded on hospital records within 30 days of death** | **Yes** | 2896 | 2003 | 4899 |
|  | **No** | 1659 | 4257 | 5916 |
|  | **Total** | 4555 | 6260 | 10815 |
| **Deaths occurring outside an admission**  **Agreement=73.2%** | | **Recorded on death certificate** | | |
|  |  | **Yes** | **No** | **Total** |
| **Recorded on hospital records within 30 days of death** | **Yes** | 479 | 541 | 1020 |
|  | **No** | 2591 | 8083 | 10674 |
|  | **Total** | 3070 | 8624 | 11694 |

**RTI-related mortality rates according to amount of information used from death certificates and hospital records**

Table S3. Number of deaths and mortality rates for respiratory tract infections and all respiratory conditions by age group 2001-2010

|  | **Respiratory tract infections *n* (rate/100,000 population)** | | |
| --- | --- | --- | --- |
| **Age group** | **Underlying cause** | **Any causes on DC^a^** | **DC+hospital admission records≤30 days before death** |
| **28-364 days** | 446 (7.2) | 950 (15.4) | 1512 (24.5) |
| **1-4 years** | 327 (1.4) | 948 (4.0) | 1411 (5.9) |
| **5-18 years** | 302 (0.3) | 1441 (1.6) | 2116 (2.4) |
|  | **Any respiratory condition *n* (rate/100,000 population)** | | |
| **Age group** | **Underlying cause** | **Any causes on DC^a^** | **DC+hospital admission records≤30 days before death** |
| **28-364 days^*^** | 1540 (25.0) | 3114 (50.5) | 4213 (68.4) |
| **1-4 years^†^** | 572 (2.4) | 1615 (6.7) | 2129 (8.9) |
| **5-18 years^‡^** | 982 (1.1) | 2896 (3.3) | 3821 (3.9) |

^a^DC=death certificate

**^*^**Total child years at risk: 6164440

^†^Total child years at risk: 23939346

^‡^Total child years at risk: 87965703

**Results of sensitivity analyses**

The difference when classifying week 26 to week 33 2009 in the winter period was less than two excess deaths per year in any age group, independent of the definition used.

Increasing the number of RTI deaths in either winter or summer by 1.8% to take into account the children aged 28 to 364 days apparently missing from the dataset altered the number of excess deaths by less than three deaths per year.

**References**

1. Fleming, DM, Pannell, RS, Cross, KW. Mortality in children from influenza and respiratory syncytial virus. J Epidemiol Community Health 2005;**59**:586-90.

2. Jansen, AGSC, Sanders, EAM, Wallinga, J, et al. Rate-difference method proved satisfactory in estimating the influenza burden in primary care visits. J Clin Epidemiol 2008;**61**:803-12.

3. Sachedina, N, Donaldson, LJ. Paediatric mortality related to pandemic influenza A H1N1 infection in England: an observational population-based study. Lancet 2010;**376**:1846-52.
